# Excess Heat Production by the Pair Annihilation of Ionic Vacancies in Copper Redox Reactions

**DOI:** 10.1038/s41598-019-49310-x

**Published:** 2019-09-23

**Authors:** Makoto Miura, Atsushi Sugiyama, Yoshinobu Oshikiri, Ryoichi Morimoto, Iwao Mogi, Miki Miura, Satoshi Takagi, Jeonghun Kim, Yusuke Yamauchi, Ryoichi Aogaki

**Affiliations:** 1 Hokkaido Polytechnic College, Otaru, Hokkaido 047-0292 Japan; 2 Yoshino Denka Kogyo, Inc., Yoshikawa, Saitama 342-0008 Japan; 3 Research Organization for Nano and Life Innovation, Waseda University, Shinjuku, Tokyo 162-0041 Japan; 4 International Center for Materials Nanoarchitectonics (WPI-MANA), National Institute for Materials Science (NIMS), Tsukuba, Ibaraki 305-0044 Japan; 5 Yamagata College of Industry and Technology, Matsuei, Yamagata 990-2473 Japan; 6 Saitama Industrial Technology Center, Kawaguchi, Saitama 333-0844 Japan; 7 Institute for Materials Research, Tohoku University, Aoba, Sendai 980-8577 Japan; 8 Polytechnic Center Kimitsu, Kimitsu, Chiba 299-1142 Japan; 9 Graduate School of Symbiotic Systems Science and Technology, Fukushima University, Fukushima, 960-1296 Japan; 10 School of Chemical Engineering and Australian Institute for Bioengineering and Nanotechnology (AIBN), The University of Queensland, Brisbane, QLD 4072 Australia; 11 Polytechnic University, Sumida, Tokyo 130-0026 Japan

**Keywords:** Chemistry, Materials chemistry

## Abstract

In the pair annihilation of ionic vacancies with opposite charges, a drastic excess heat production up to 410 kJ mol^−1^ in average at 10 T (i. e., 1.5 times larger than the heat production by the combustion of H_2_,  285.8 kJ mol^−1^) was observed, which was then attributed to the emission of the solvation energy stored in 0.61 nm radius vacancies with two unit charges. Under a high magnetic field, using Lorentz force, we made ionic vacancies created in copper cathodic and anodic reactions collide with each other, and measured the reaction heat by their annihilation. Ionic vacancy is initially created as a byproduct in electrode reaction in keeping the conservation of linear momentum and electric charge during electron transfer. The unstable polarized particle is stabilized by solvation, and the solvation energy is stored in the free space of the order of 0.1 nm surrounded by oppositely charged ionic cloud. The collision of the ionic vacancies was carried out by circulation-type magnetohydrodynamic electrode (c-type MHDE) composed of a rectangular channel with a pair of copper electrodes and a narrow electrolysis cell.

## Introduction

Hydrogen and oxygen gas bubble evolution by water electrolysis is one of the popular classroom demonstrations of vigorous electrochemical processes^1–3^. In this experiment, the electrode potentials of cathode and anode must exceed the hydrogen and oxygen evolution potentials, respectively. However, in various electrode reactions under high magnetic fields, without exceeding the hydrogen and oxygen evolution potentials, a large amount of microbubbles containing dissolved nitrogen gas have been observed^4–6^.

These experimental results strongly suggest that ionic vacancy is generally created as a byproduct in electrode reaction. From the theoretical and experimental examinations, the following natures of ionic vacancy have been clarified; ionic vacancy in steady state is an electrically polarized free space of the order of 0.1 nm (vacancy core) surrounded by ionic cloud with opposite charge^7^, which is initially formed in keeping the conservation of linear momentum and electric charge during electron transfer^8^. The amount and sign of the polarized charge are equal to those of the charge transferring in electrode reaction. Since an electric charge isolated in solvent is unstable, ions are immediately stabilized by solvation, emitting solvation energies with entropy production. In the case of ionic vacancy, using the solvation energy, the initially formed vacancy core grows up to a size of the order of 0.1 nm without entropy production^7^. Namely, the solvation energy is stored in vacancy core. The lifetime has been determined about 1 second^9^, so that ionic vacancies with the same charge have sufficient times to form nanobubble by collision^10^. The microbubbles observed were therefore attributed to the coalescence of the nanobubbles.

Chemical nature of ionic vacancy has been examined by the adsorption onto newly created copper nuclei, which, though no hydrogen evolution, resulted in obvious dendritic growth (Magneto-dendrite effect^11^). This implies that instead of proton, ionic vacancy specifically adsorbs on copper surface, and nanobubble from ionic vacancies like hydrogen molecule from protons suppresses three-dimensional (3D) copper nucleation. The most remarkable point of this effect is that the rising deposition current greatly shifts to anodic side from the hydrogen evolution potential, so that in a high magnetic field, copper cathodic deposition proceeds without hydrogen evolution even in a current much larger than the limiting-diffusion current.

However, the reactivity of ionic vacancy has not yet been fully clarified. As have been mentioned above, the collision between ionic vacancies with the same sign yields nanobubbles. On the other hand, in case of the collision between ionic vacancies with opposite signs, what brings about? Due to charge neutralization of ionic clouds, the expected answer would be the annihilation of the vacancies. Since there is no reaction product, how to detect such a reaction is the next problem.

For the reaction analyses in complex electrochemical cells such as lithium batteries, calorimetry experiments are quite useful^12,13^. Here, in order to exactly measure the quantity of heat, it is indispensable to keep electrode systems isothermal. Though such a condition is often interfered by the nonuniform temperature fields occurring in electrode systems, we can fortunately find a good solution in magnetoelectrochemical devices. In magnetoelectrochemistry, some useful tools have been provided for the reaction analyses in magnetic field^14–21^.

In magnetically assisted electrolysis under a magnetic field parallel to electrode surface, Lorentz force induces a solution flow called magnetohydrodynamic (MHD) flow promoting mass transport of ions. An application of the MHD flow in a parallel magnetic field led to the development of MHD-pumping electrode cell called MHD electrode (MHDE)^22–24^, where the velocity and concentration distributions reduce to the simple equations of the velocity and limiting diffusion current. In MHDE, agreement between theory and experimental result is excellent^18^. A notable advantage of magnetically excited solution flow therefore lies in the practical possibility of using very small cells without mechanical means. Especially, the strong stirring by Lorentz force in high magnetic field easily attains isothermal condition.

In the present paper, therefore, using positive and negative ionic vacancies created by copper cathodic and anodic reactions in a MHDE cell specially designed, we measure the reaction heat of a pair of ionic vacancies with opposite signs.

## Results

### Theory

**Figure 1:**
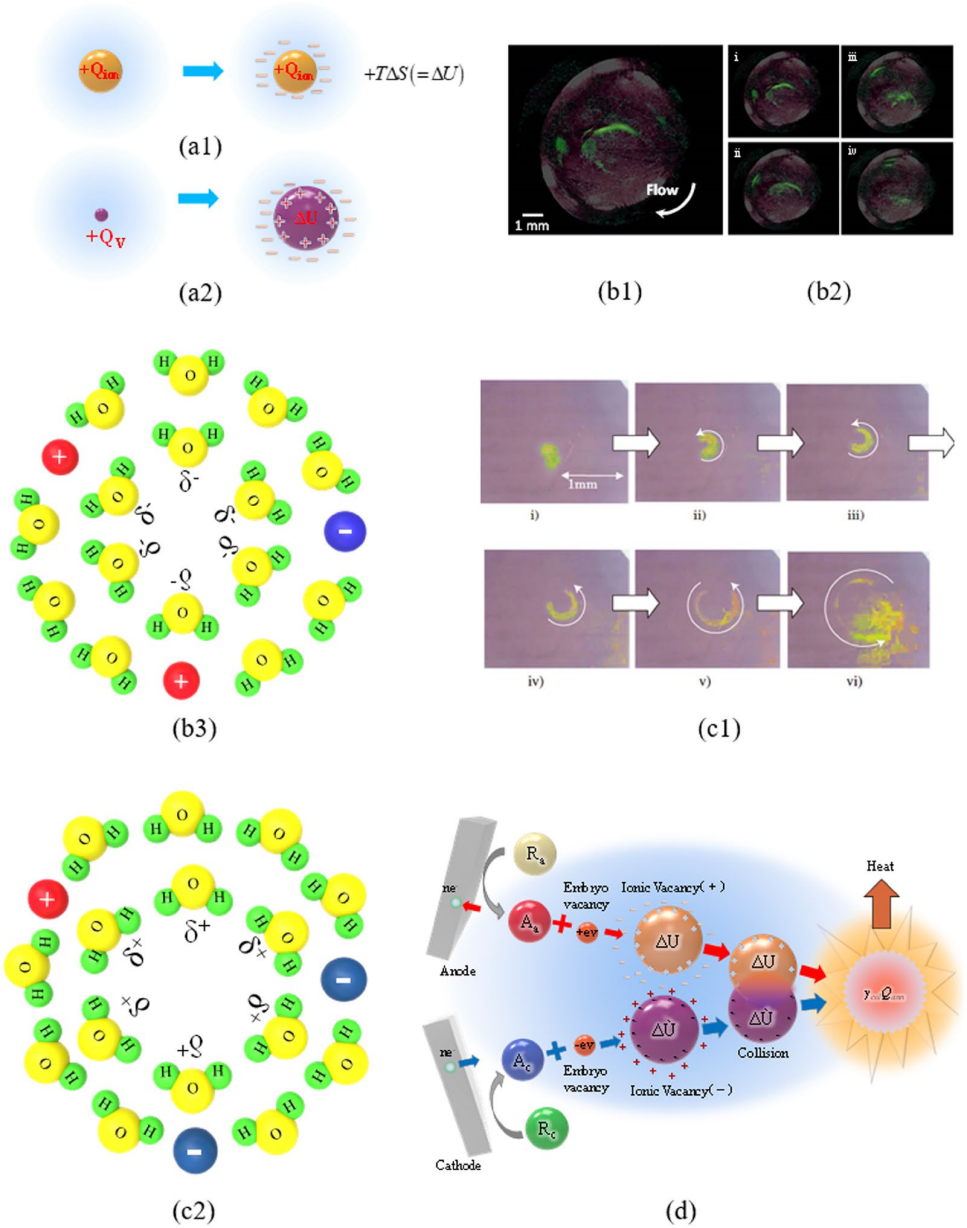
Creation and pair annihilation of ionic vacancies with opposite charges. (**a1**) Solvation of ion (in case of anion): Entropy is produced by the solvation energy emitted from the ionic cloud. +*Q*_*ion*_, Ionic charge of anion; *ΔS*, Produced  entropy; *T*, Absolute temperature. (**a2**) Solvation of positive ionic vacancy: Instead of entropy production, the vacancy core is expanded by the solvation energy, i.e., it is stored in the vacancy core. +*Q*_*V*_, Polarized charge of positive vacancy; *ΔU*, Solvation energy stored in vacancy core. (**b1**) Microbubble evolution in copper cathodic deposition under a tornado-like vortex called vertical MHD flow at 8 T (Modified^5^). [CuSO_4_], 30 mol m^−3^; [H_2_SO_4_], 100 mol m^−3^. Electrode potential, *V* = −144 mV (+125 mV vs. NHE). For clear visualization, the images of the globules are painted in green. (**b2**) Continuous images of the microbubble motion at a 70 ms interval. The observed angular velocity of MHD flow was 6.67 s^−1^, which is shown in the form of arrow in (**b1**). For clear visualization, the images of the globules are painted in green. (**b3**) Schematic of a vacancy core with negative polarized charge formed in water solution. H, Proton; O, Oxygen atom; δ^−^, Partial polarized charge of the water molecule surrounding the free space; ⊖, Cation: ⊕, Anion. (**c1**) Continuous images of microbubble evolution in copper anodic dissolution under a vertical MHD flow at 8 T, which were taken at a 0.33 s interval (Modified^6^). *V* = +150 mV (+400 mV vs. NHE); [CuSO_4_], 30 mol m^−3^; [H_2_SO_4_], 100 mol m^−3^. The images of the microbubbles are painted in yellow. (**c2**) Schematic of a vacancy core with positive polarized charge formed in water solution. H, Proton; O, Oxygen atom; δ^+^, Partial polarized charge of the water molecule surrounding the free space; ⊖, Cation: ⊕, Anion. (**d**) Pair annihilation of positive and negative vacancies. ne^-^, transferring electric charge of electrons; A_a_, Anodic activated complex; A_c_, Cathodic activated complex; +ev, Positive embryo vacancy; −ev, Negative embryo vacancy; *ΔU*, Stored solvation energy; γ_col_Q_ann_, Molar excess heat.

Figure 1a represents the comparison of the solvation processes between ion and ionic vacancy. Since a single electric charge isolated in solution is unstable, it is stabilized by forming ionic cloud, emitting solvation energy. In case of ion, the solvation energy is emitted outside, producing entropy, whereas ionic vacancy expands by using the solvation energy, storing it in the vacancy core. Figure 1b,c exhibits the microbubbles observed in the copper cathodic and anodic reactions and the structures of the vacancy cores storing solvation energies. The microbubbles arise from the collision of nanobubbles, and the nanobubbles come from the collision of the solvated vacancies of the same charge.

The equilibrium potential of copper cathodic deposition is more anodic than the hydrogen evolution potential, while copper anodic dissolution occurs at an equilibrium potential more cathodic than the oxygen evolution potential. Therefore, the cathodic and anodic reactions in acidic cupric sulfate solution involving vacancy production are simply expressed as follows,

1 $${{\rm{Cu}}}^{2+}+{{\rm{2e}}}^{-}\to {\rm{Cu}}+{{\rm{V}}}_{2-}\,({\rm{Cathodic}}\,{\rm{reaction}})$$

2 $${\rm{Cu}}-2{{\rm{e}}}^{-}\to {{\rm{Cu}}}^{2+}+{{\rm{V}}}_{2+}\,({\rm{Anodic}}\,{\rm{reaction}})$$

where V_2−_ and V_2+_ depict the vacancies with −2 and +2 charges, respectively. In the isolated state of stationary solution, whether the charges are the same or not, individual ionic vacancies reversibly disappear without any heat production. However, in case of the collision of the vacancies with opposite charges, as shown in Fig. 1d, the neutralization of ionic clouds would lead to the following pair annihilation,

3 $${{\rm{V}}}_{2+}+{{\rm{V}}}_{2-}\to {\rm{N}}{\rm{u}}{\rm{l}}{\rm{l}}+{\gamma }_{col}{Q}_{ann}$$

where *γ*_*col*_ is the collision efficiency, and *Q*_*ann*_ is the molar excess heat (J mol^−1^) by the annihilation. As have been discussed above^7,8^, by the thermal motion of solution particles, an initial embryo vacancy emitted in solution phase is quickly stabilized by solvation to thermodynamic equilibrium, where the solvation energy is stored in the vacancy core by the expansion work of the free-space sphere (Fig. 1a).

In a quasi-static reversible process, the expansion work is simply expressed by

4 $$d{w}_{vacancy}=-\,\Delta \,pdv$$

where *v* is the volume of the vacancy core, i.e.,

5 $$v=\frac{4}{3}\pi {r}^{3}$$

where *r* is the radius. Δ*p* is the pressure difference between the external and internal pressures, which arises from the surface tension *σ* of the inner wall of vacancy, referred as the Young-Laplace equation,

6 $$\Delta p=-\,\frac{2\sigma }{r}$$

As have been discussed in the previous paper^7^, it is not changed by the polarization of the inner wall, and equalized to the surface tension of pure water even in the presence of electrolyte. Substituting Eqs. (5) and (6) into Eq. (4), and integrating the resulting equation from *r* = 0 to *r* = R^*^, we obtain the vacancy formation work as the increment of the internal energy of the vacancy.

7 $$\Delta {U}_{vacancy}=4\pi \sigma {R}^{\ast 2}$$

where R^*^ is the radius of steady-state vacancy core. Adding Boltzmann energy (3/2)*k*_*B*_*T* (*k*_*B*_ is Boltzmann constant, and *T* is absolute temperature) and polarization energy of the inner wall, we obtain the total internal energy of ionic vacancy. On the other hand, transferring electron loses the same amount of kinetic energy, so the molar heat production of the system by the pair annihilation is given by

8 $${Q}_{ann}=8\pi {N}_{A}\sigma {R}^{\ast 2}$$

where *N*_*A*_ is the Avogadro number. Inserting the data R^*^ = 0.5 to 1.0 nm with *N*_A_ = 6.0 × 10^23^ mol^−1^ and *σ* = 7.2 × 10^−2^ J m^−2^ (surface tension of water at 25 °C) into Eq. (8), we obtain the liberated molar excess heat as *Q*_*ann*_ = 271 to 1086 kJ mol^−1^, i.e., the excess heat production by the pair annihilation is estimated of several 100 kJ mol^−1^. Equation (8) indicates that the larger the radius of vacancy core is, the larger the molar excess heat is. As discussed in the previous paper^7^, the radius is approximately proportional to the vacancy charge, so that the collision of the vacancies with two unit charges in the present case would yield much larger excess heat than the collision of the vacancy with a single unit charge.

**Figure 2:**
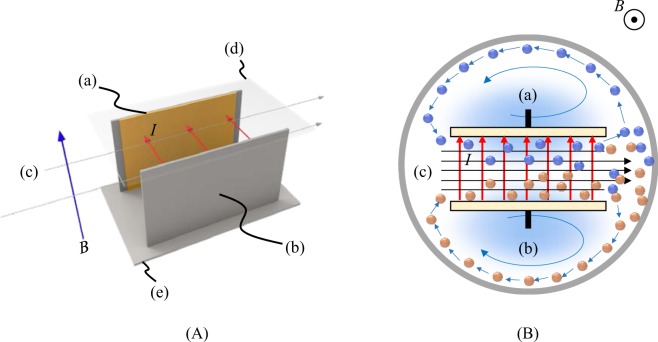
Schematic view of the c-type MHDE. (**A**) Bird’s-eye view, (**B**) Horizontal cross section. (a) Cathode, (b) Anode, (c) Streamlines, (d) Upper wall, (e) Bottom wall. *I*, Electrolytic current; *B*, Magnetic flux density; blue circle, Negative vacancy; orange circle, Positive vacancy.

Figure 2 shows the circulation-type MHDE (c-type MHDE), which is composed of a rectangular channel with two open ends and a pair of copper electrodes imbedded face to face as cathode and anode on the inner walls, being settled in a narrow electrolysis cell. In accordance with Eqs. (1) and (2), vacancies created on the electrode surfaces with opposite charges are conveyed by the MHD flow, and hydrodynamically mingled to collide at the front wall of the electrolysis cell. Due to narrow space, the vacancies escaping from the collision quickly circulate with the electrolyte solution, as will be discussed later, contributing to the suppression of hydrogen and oxygen evolutions.

### *Currentsweepmethodforcalorimetryexperiment*

To calculate the reaction heat by the pair annihilation, we first assume a positive current *I* (A) increasing with time *t* (s).

9 $$I=at\,{\rm{for}}\,t\ge 0$$

where *a* is the positive sweep rate (A s^−1^). The current sweep yields two regions of the cell voltage. Initially, due to small current, Lorentz force is weak, so that the slow solution flow makes the cell voltage Δ*V*_1_ (V) approximately change with the concentration overpotential Δ*H* (V) involving cathodic and anodic overpotentials, i.e.

10 $$\Delta {V}_{1}\approx \Delta H$$

Then, the Lorentz force increasing with the current *I* gradually accelerates the solution flow, and resultantly suppresses Δ*H*. After attaining a critical current *I*_*C*_, instead of Δ*H*, the second cell voltage Δ*V*_2_ (V) emerges, which is effectively expressed by the reaction overpotential and ohmic drop, i.e., Δ*V*_2_ linearly increases with *I*.

11 $$\Delta {V}_{2}=\Delta {V}_{react}+{R}_{sol}I\,\,{\rm{for}}\,I\ge {I}_{C}$$

where Δ*V*_*react*_(V) and *R*_*sol*_(Ω) are the reaction cell voltage and the solution resistance, respectively.

Using the relationship *dI* = *adt* in Eq. (9), integrating Eq. (A28) in Supplement A with respect to *I*, we obtain the total heat production, *Q*_*total*_ (J).

12 $${C}_{sys}(\Delta {T}^{\ast }-\Delta {T}_{0}^{\ast })(={Q}_{total})=\frac{1}{a}{\int }_{0}^{I}\Delta VIdI+\frac{-T{\Delta }_{R}{S}_{R}+{\gamma }_{col}{Q}_{ann}}{2nFa}{I}^{2}$$

where *C*_*sys*_ (J K^−1^) is the calorimeter constant, ΔT^*^ is the compensated total temperature difference (K), and $\varDelta {T}_{0}^{\ast }$ is the initial value of ΔT^*^(s). *n* is the positive charge number tranferring in the cell reaction, and *F* is Faraday constant (96500 C mol^−1^). *T* is the absolute temperature of the system (K), Δ_*R*_*S*_*R*_ is the molar entropy of the cell reaction (J K^−1^ mol^−1^), and *Q*_*ann*_ is the molar excess heat of vacancy (J mol^−1^), and *γ*_*col*_ is the collision efficiency. The first term on the right hand side in Eq. (12) is the Joule’s heat *Q*_*Joule*_, i.e.,

13 $${Q}_{Joule}=\frac{1}{a}{\int }_{0}^{I}\Delta VIdI\,({\rm{J}}\,{{\rm{mol}}}^{-1})$$

Equation (13) cannot be applied to linear regression as it is. To integrate the product of the cell voltage and current Δ*VI* with regard to *I*, Eq. (11) is substituted into Eq. (13). The integral beyond the critical current *I*_*C*_ is simply expressed by

14 $${\int }_{0}^{I}\Delta VIdI={\int }_{0}^{{I}_{C}}\Delta {V}_{1}IdI+\frac{\Delta {V}_{react}}{2}({I}^{2}-{I}_{C}^{2})+\frac{{R}_{sol}}{3}({I}^{3}-{I}_{C}^{3})$$

Inserting Eq. (14) into Eq. (12), we obtain the following third order equation of *I*.

15 $$\Delta {T}^{\ast }={a}_{0}+{a}_{2}{I}^{2}+{a}_{3}{I}^{3}\,{\rm{for}}\,I\ge {I}_{C}$$

where

16 $${a}_{3}=\frac{{R}_{sol}}{3a{C}_{sys}}$$

17 $${a}_{2}=\frac{1}{2a{C}_{sys}}(\Delta {V}_{react}+\frac{-T{\Delta }_{R}{S}_{R}+{\gamma }_{col}{Q}_{ann}}{nF})$$

18 $${a}_{0}=\frac{1}{a{C}_{sys}}({\int }_{0}^{{I}_{C}}\Delta {V}_{1}IdI-\frac{\Delta {V}_{react}}{2}{I}_{C}^{2}+\frac{{R}_{sol}}{3}{I}_{C}^{3})+\Delta {T}_{0}^{\ast }$$

After analyzing the linear regression by Eq. (15) for the seccond range of the cell voltage Δ*V*_2_, the calorimeter constant *C*_*sys*_ is determined by *a*_3_ in Eq. (16),

19 $${C}_{sys}=\frac{{R}_{sol}}{3a{a}_{3}}$$

and the observed molar excess heat *γ*_*col*_*Q*_*ann*_ is obtained from *a*_2_ in Eq. (17).

20 $${\gamma }_{col}{Q}_{ann}=nF(2a{a}_{2}{C}_{sys}-\Delta {V}_{react})+T{\Delta }_{R}{S}_{R}$$

For a redox cell reaction such as the present case, due to no chemical production, *T*Δ_*R*_*S*_*R*_ = 0 is assumed.

The constant term *a*_0_ in Eq. (18) is yielded by the initial Joule’s heat generation up to *I*_*C*_. Due to insufficient stirring by weak Lorentz force, the accuracy of this term is much lower than other terms.

### *Excess heatevolution in Cu/CuSO*_*4*_*electrochemicalsystem*

**Figure 3:**
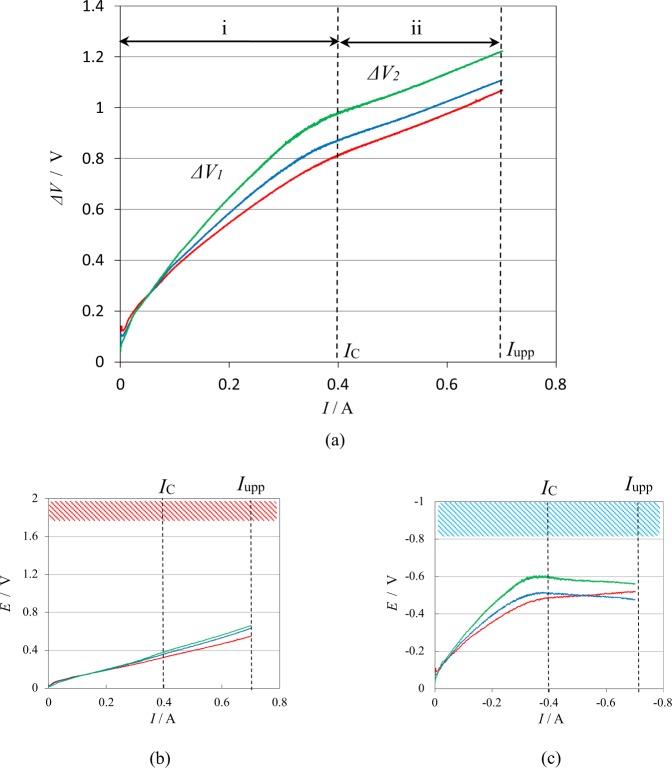
Cell voltage responses against the current sweep in copper redox reactions under high magnetic fields. (**a**) Cell voltage response against the current sweep in a rate of 0.2 mA s^−1^. Green line, at 5 T; Blue line, at 10 T; Red line, at 15 T. (**b**) Anodic potential response against the same current sweep. Green line, at 5 T; Blue line, at 10 T; Red line, at 15 T. (**c**) Cathodic potential response against the same current sweep. Green line, at 5 T; Blue line, at 10 T; Red line, at 15 T. i, Initial voltage range *ΔV*_*1*_; ii, Linear voltage range *ΔV*_*2*_; *I*_*c*_, Critical current; *I*_*upp*_, Upper limit of the sweeping current. Red and blue zones denote the oxygen and hydrogen evolution areas, respectively.

Figure 3a represents the response of the cell voltage Δ*V* against the sweeping current *I* in a rate of 0.2 mA s^−1^ up to an upper limit of current *I*_*upp*_ =  0.7 A at 5 T, 10 T and 15 T. From these results, the critical current *I*_*C*_ = 0.4 A was determined. From the linear relationship between Δ*V*_2_ and *I* in Eq. (11), the solution resistance *R*_*sol*_ and the reaction cell voltage Δ*V*_*react*_ were calculated for each measurement. The average values are *R*_*sol*_ = 1.01 ± 0.30 Ω and Δ*V*_*react*_ = 0.513 ± 0.133 V. To ascertain that hydrogen- and oxygen-gas evolution reactions are not involved in the range from 0.4 A to 0.7 A, in Fig. 3b,c, individual potential responses of the anode and cathode are plotted. For comparison, oxygen evolution area (beyond 1.8 V) and hydrogen evolution area (beyond −0.8 V) emerging in the current sweep without magnetic field are exhibited; neither of the potentials exceeds the gas-evolution potentials, i.e., there is no gas evolution.

In Fig. 3b, the anodic potential linearly increases with the current, and takes lower values than the oxygen-evolution potential (1.8 V) until 0.7 A. In zero-magnetic field, however, around 0.4 A, the potential quickly transferred to 1.8 V. This result implies that nanobubbles arising from the vacancy layer on the anode strongly suppress oxygen gas evolution by the adsorption on the active sites of the electrode. In case of copper cathodic deposition in Fig. 3c, the situation for the reaction without gas evolution is more severe; the reaction is restricted by the limiting-diffusion current of copper ions, so that at higher currents, hydrogen gas may inevitably evolved. Actually, in the absence of magnetic field, about at −0.1 A, the potential shifted to the hydrogen evolution potential (−0.8 V). However, as have been discussed initially, based on the magneto-dendrite effect^11^, the dendritic growth by nanobubble adsorption from vacancies allows us for much larger current to flow without hydrogen-gas evolution. As shown in Fig. 3c, initially, the potentials increase with the current. After passing −0.4 A, they take almost constant values around −0.4 V. Due to these potential behaviors, the linear cell-voltage relationship of Δ*V*_2_ shown in Fig. 3a is attributed to such a difference between the anodic and cathodic potential responses.

**Figure 4:**
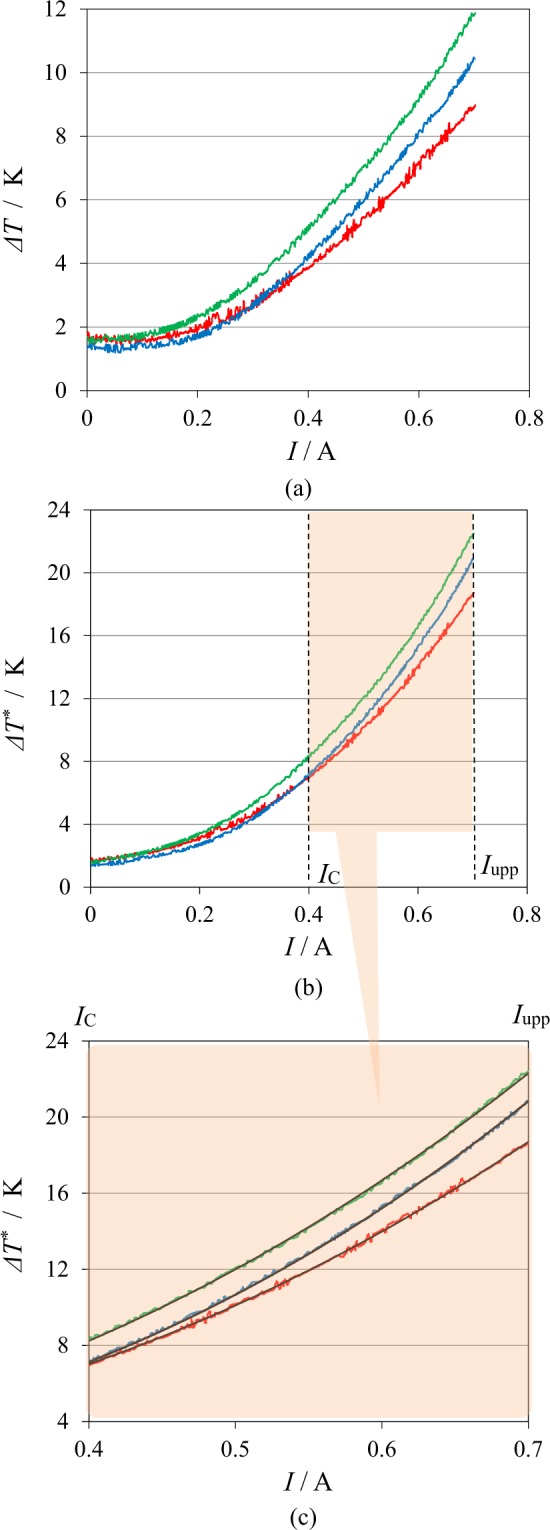
Temperature responses against the current sweeps under high magnetic fields. (**a**) Direct temperature difference *ΔT* measured during experiment vs. current *I* plot. (**b**) Compensated temperature difference ΔT^*^ vs. current *I* plot. (**c**) Comparison of the measured data with the linear-regression data. Current sweep rate is 0.2 mA s^−1^. Green line, data measured at 5 T; Blue line, at 10 T; Red line, at 15 T. Black line is the linear-regression data, which was applied to the current range between the critical current *I*_*c*_ and the upper limit *I*_*upp*_.

In Fig. 4a, the temperature difference Δ*T* between the solution and the environment measured during experiment are directly plotted against current *I*. Under a magnetic field of 10 T, it increases up to 10.5 K, i.e., Δ*T*≈ 10.5 K at the upper limit of 0.7 A. However, the temperature difference decreases with the heat escaping from the cell system, so that to determine the amount of the escaping heat per unit time, the solution temperature was measured by switching off the current *I* after reaching the upper limit. Following Eq. (A24) in Supplement A, the temperature difference from the ambient temperature was plotted against time in semi-log scale. From the slopes of the plot, the time constant *α* was calculated for each measurement. The average value was *α* = 7.24 × 10^−4^ ± 9.8 × 10^−5^ s^−1^. Using the measured temperature difference Δ*T* and each value of *α*, in accordance with Eq. (A27) in Supplement A, we determined the compensated temperature difference ΔT^*^ with the increasing current *I* at a given magnetic flux density *B*. In Fig. 4b, ΔT^*^ was plotted against *I*. Then, the least squares of Eq. (15) were applied to the experimental data between 0.4 A and 0.7 A. Figure 4c represents a result of the linear regression, where the linear-regression data are expressed in black lines, which are extremely consistent with the experimental data. After ascertaining the good agreement with the measured data, for each measurement, the calorimeter constant *C*_*sys*_ and the observed molar excess heat *γ*_*col*_*Q*_*ann*_ were individually calculated. The average value of the calorimeter constant is *C*_*sys*_ = 1.50 × 10^2^ ± 7.5 × 10 J K^−1^.

**Figure 5:**
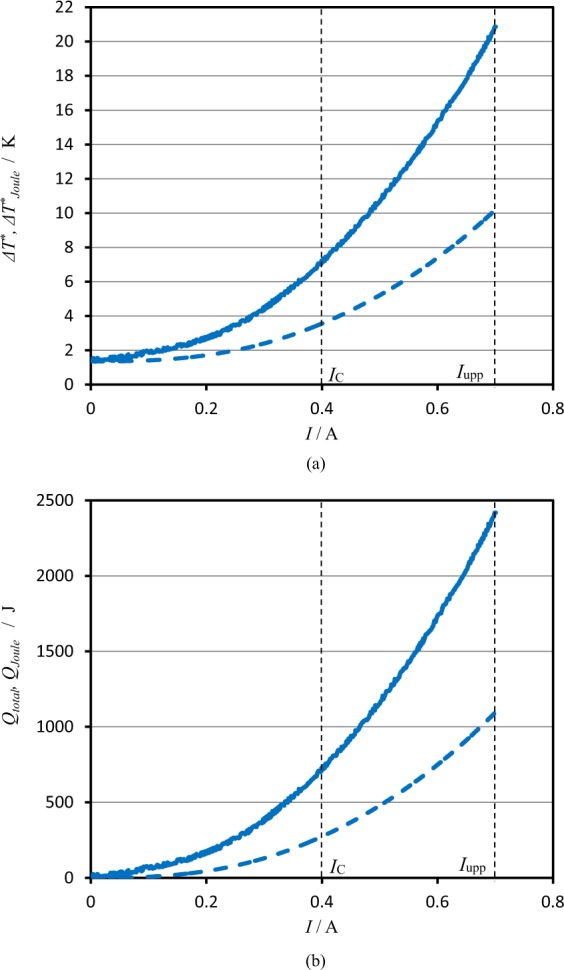
Contribution of Joule’s heat to the total heat production at 10 T. (**a**) Plots of the compensated temperature difference vs. current sweep. Solid line is the total compensated temperature difference ΔT^*^, and break line is the contribution of Joule’s heat ΔT^*^_Joule_. (**b**) Plots of heat production vs. current sweep. Solid line is the total heat production *Q*_*total*_, whereas the break line is the Joule’s heat production *Q*_*Joule*_. Current sweep rate is 0.2 mA s^−1^. The calorimeter constant *C*_*sys*_ in this case is 124 J K^−1^. *I*_*c*_, the critical current (0.4 A); *I*_*upp*_, the upper limit of the current (0.7 A). Linear regression was applied to the area between *I*_*c*_ and *I*_*upp*_.

Figure 5a represents the plot of the compensated total temperature difference ΔT^*^ vs. current *I*. For comparison, the compensated temperature difference $\Delta {T}_{Joule}^{\ast }$ induced by the Joule’s heat is also plotted. At 0.7 A, the total temperature difference attains 21 K, i. e., ΔT^*^ ≈ 21 K, where the contribution of the Joule’s heat is only 48%, i.e., $\Delta {T}_{Joule}^{\ast }$≈ 10 K. In Fig. 5b, by using the obtained calorimeter constant *C*_*sys*_, the total heat production *Q*_*total*_(=*C*_*sys*_ΔT^*^) and the Joule’s heat *Q*_*Joule*_ are exhibited. The difference between them corresponds to the observed excess heat. In the initial current area up to 0.1 A, the contribution of Joule’s heat is neglected, whereas at the final stage of 0.7 A, the Joule’s heat component attains 48% of the total heat amount. This is because the cell voltage increases with the current sweep.

**Figure 6:**
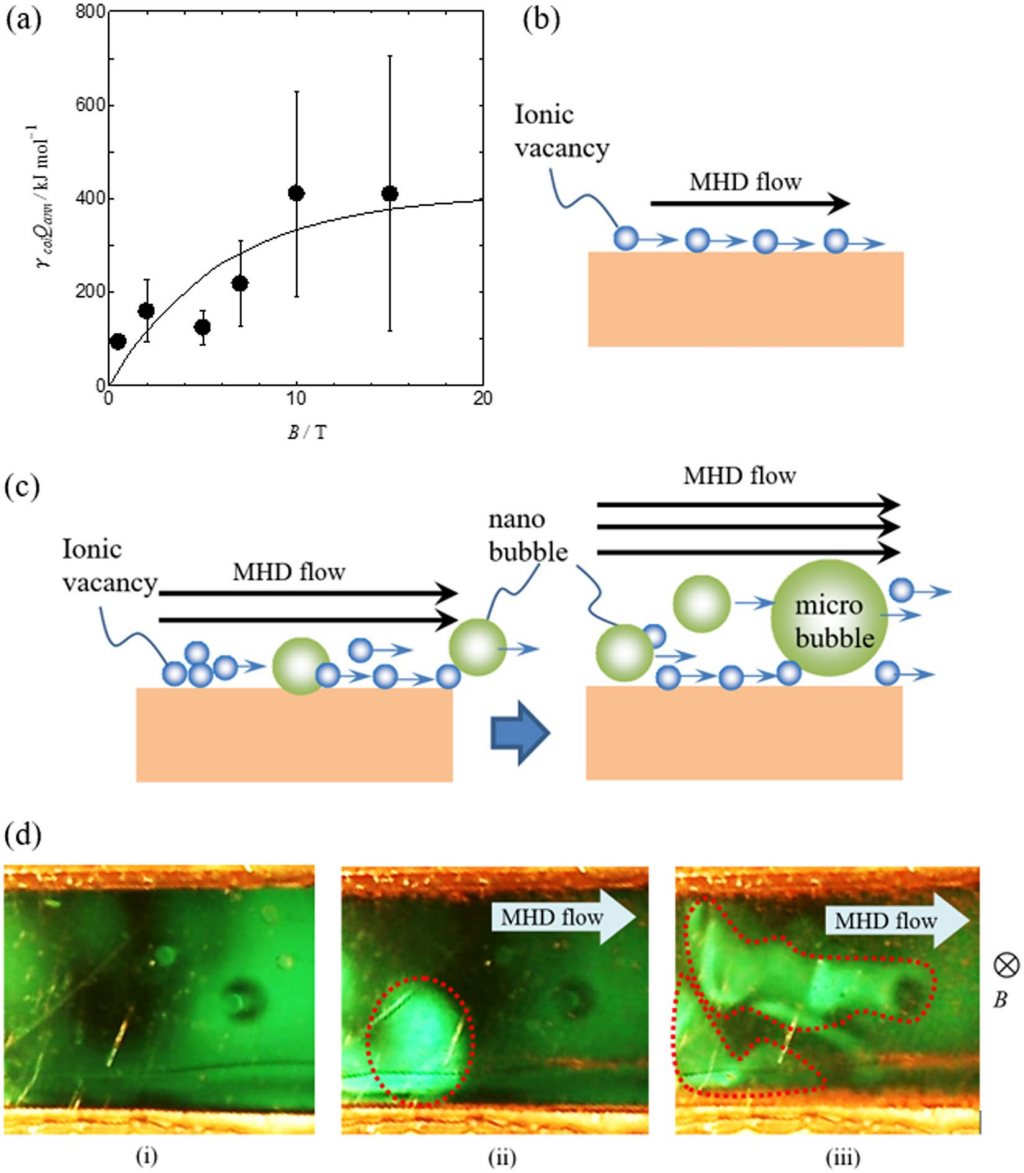
The dependence of the observed molar excess heat on magnetic flux density. (**a**) Plot of the observed molar excess heat *γ*_*col*_*Q*_*ann*_ vs. magnetic flux density *B*. At almost all magnetic flux densities, more than four times measurements were performed. (**b**) In a low magnetic field, ionic vacancies are smoothly conveyed along the electrode by laminar MHD flow. (**c**) In a high magnetic field, ionic vacancies collide with each other on the electrode by accelerated MHD flow, yielding nanobubbles and microbubbles, and disturbing the collision probability of ionic vacancies. (**d**) Microbubble clusters observed in the MHD flow of c-type MHDE at 5 T. (i) *I* = 0 A, (ii) *I* = 0.225 A, (iii) *I* = 0.385 A. White areas encircled by dotted lines are microbubble clusters.

In Fig. 6a, the observed molar excess heats are plotted against magnetic flux densities. The observed excess heat converges to zero with decreasing magnetic flux density, i.e., the vacancy collision does not occur in stationary solution (*γ*_*col*_ = 0). On the other hand, as the magnetic field increases, the values of the observed amounts of heat are widely scattered, and gradually reach the highest limit of ca. 410 kJ mol^−1^ in average. When magnetic field is not so high, after smoothly conveyed along the electrode surface by laminar MHD flow, ionic vacancies collide with each other in the bulk solution (Fig. 6b). As a result, the excess heat production increases with increasing magnetic field. However, as shown in Fig. 6c, with magnetic field, Lorentz force increases, so that MHD flow also increases the velocity, which enhances nanobubble formation by the collision of vacancies with the same charge, reducing the concentration of ionic vacancy. Such a promoted fluid flow accelerates further collisions of the nanobubbles, giving rise to microbubbles. Figure 6d exhibits the clusters of microbubbles occurring in the MHD flow of c-type MHDE at 5 T. The microbubbles evolved greatly disturb the chance of collision, leading to the wide scattering of the data at the higher magnetic fields. As magnetic flux density increases, stronger MHD flow arises from stronger Lorentz force. As a result, the slight change in the configuration of MHD electrode together with the enhanced formation of microbubble induces the instability of the flow, which brings about very large error bar ranges at 10 T and 15 T in Fig. 6a.

## Discussion

As shown in Supplement A, an applied magnetic field induces MHD flow. Energy of matter activated by a 10 T magnetic field is only of the order of 1 milli eV, so that the almost all energy increment by the magnetic field comes from the motion of MHD flow. The enhanced energies of MHD flow are classified into kinetic energy of the flow, dissipated friction heat^25^ and the work of the pressure difference between the outlet and inlet of MHDE, which are, however, about one-thousandth times smaller than the Joule’s heat production. As discussed in Supplement A, the reversible heat generated at the electrode/electrolyte junction by electrochemical reaction arises from the change in partial molar entropy of the half-cell reaction plus terms which relate to the interaction between heat and mass transport in the electrode (i.e., the thermocouple or electronic Seebeck effect) and the electrolyte (i.e., the thermal diffusion or Soret effect)^26,27^. However, in the present case, the electric transport-related terms are cancelled since the same electrode and metal lead are used in each half cell^28^, and due to the isothermal condition maintained by MHD flow, the electrolytic transport-retated terms are disregarded. As a result, the reversible heat for the whole cell reaction can be calculated simply from the entropy change of reaction, i.e., *T*Δ_*R*_*S*_*R*_^13^. In the present case of copper redox reactions, the half cell reactions in Eqs. (1) and (2) cancel their reversible heats, so that *T*Δ_*R*_*S*_*R*_ = 0 is concluded. Namely, if the vacancy annihilation were neglected, heat production except for Joule’s heat would not be observed.

In the calorimetry experiment of the copper redox reactions in copper sulfate solution without hydrogen and oxygen evolution by using the MHD flow in a copper c-type MHDE, we observed at most a 10.5 °C (10.5 K) temperature increase of the system, which was translated into 21 °C (21 K) by the compensation of the escaping heat from it. The contribution of Joule’s heat to the total temperature increase was less than 48%. Then, by using linear regression, from the experimental data, the observed molar excess heat production was determined. The heat production results from the collision of a pair of ionic vacancies with opposite charges, and attains an uppermost value of 410 kJ mol^−1^ at 10 T in average, which was 1.5 times larger than the heat production by the combustion of hydrogen molecule 285.8 kJ mol^−1^ at 25 °C, 1 bar. On the contrary, as the magnetic field decreased to 0 T without MHD flow, the measured excess heat converged to zero. As mentioned above, this result validates that the excess heat production comes from the vacancy collision by MHD flow. Assuming that the collision efficiency *γ*_*col*_ attains almost 1.0 at 10 T, we can evaluate the vacancy radius corresponding to the uppermost value of 410 kJ mol^−1^, i.e., from Eq. (8), R^*^ = 0.61 nm is obtained, which is in good agreement with the theoretical value 0.75 nm (corresponding to ${Q}_{ann}$ = 610 kJ mol^−1^) obtained in the previous paper^7^. The radius of 0.75 nm was, however, calculated in the case of a 100 mol m^−3^ H_2_SO_4_ solution, so that for the same concentration as the present case of 500 mol m^−3^, according to the present theory, we obtain 0.72 nm corresponding to ${Q}_{ann}$ = 570 kJ mol^−1^. In addition, as have been discussed above, the large error bar ranges at 10 T and 15 T come from the instability of the MHD flow enhanced by the electrode configuration and microbubble formation.

In conclusion, the solvation energy stored in vacancy cores is liberated in the pair annihilation as excess heat by the neutralization of oppositely charged ionic clouds. This is a universal phenomenon of electrode reaction. As an example of electrode reactions, we adopted copper redox reaction because of a large amount of excess heat production by the vacancies with two unit charges. Namely, whatever the reaction is, we can expect the same kind of excess heat production. Copper plating and copper electrolysis refining are major industrial electrochemical processes. Therefore, the recycling of the wasted thermal energy of ionic vacancies would be a contemporary energy issue for global economy and ecology.

## Methods

Copper deposition was performed in a 300 mol m^−3^ CuSO_4_ + 500 mol m^−3^ H_2_SO_4_ solution. Water was prepared by a pure water production system (@ millipore filter Co.). CuSO_4_ and H_2_SO_4_ were in analytical grade (Wako pure chemical Co.). MHDE was composed of a channel of acrylic acid resin with two open ends; the channel was 10 mm high, 5 mm wide and 22 mm long. A pair of rectangular Cu electrodes (10 × 20 × 1 mm, Iwasaki Co., oxygen-free copper, 99.96% purity) working as cathode and anode were imbedded on the inner side walls. After confirming that the influence of magnetic field up to 15 T was below the environmental thermal disturbance, two thermal sensors (T-type thermocouple) were attached to the electrodes from the outside of the channel, of which leads were connected to a measuring instrument (Keyence Co., NR-600 with NR-TH08 unit). Then, the MHDE was set in the vessel containing an electrolytic solution of 7.5 cm^3^ (c-type MHDE), and the whole electrode system was settled in the bore space of 15 Tesla-cryocooled superconducting magnet at the High Field Laboratory for Superconducting Materials, Institute for Materials Research, Tohoku University. The solution flow in the MHDE was optically observed by a microscope (AnMo Electronics Co., Dino-Lite Premier2 S-DINOAD7013MT) from the bottom of the bore. Finally, other two sensors were inserted to monitor the temperatures of the solution and the bore space. After ascertaining that the sensors attached to the electrodes indicated the same temperature as that of the solution, for simplicity, they were removed from the electrodes. The two sensors in the solution and the bore space were used for measurement. Sweeping the electrolysis current *I* in a rate of 0.2 mA s^−1^ from 0 A to 0.7 A with a potentiostat (Toho Technical Research Co., Ltd., PS-2000) in galvanostatic mode, we measured the potential response Δ*V* between the cathode and anode of the c-type MHDE. The electrode potentials of cathode and anode were measured by the tentative reference electrode of a copper rod of 0.3 mm diameter. During the experiment, the temperatures of the electrodes, the solution and the bore space were measured. After attaining an upper limit of 0.7 A, to measure the heat escaping from the electrode system, the current was switched off, and decreasing temperature of the solution was recorded by a personal computer.

## Supplementary information

Supporting information
