# Supplementary material for: Excess Heat Production by the Pair Annihilation of Ionic Vacancies in Copper Redox Reactions
Source: Sci Rep. 2019 Sep 23;9:13695. doi: 10.1038/s41598-019-49310-x (PMC6757050; doi:10.1038/s41598-019-49310-x)
Supplement: Supplementary file 1 — Supporting information [file 41598_2019_49310_MOESM1_ESM.pdf]

Supplementary material for

Excess Heat Production by the Pair Annihilation of Ionic Vacancies in Copper Redox Reactions

Makoto Miura[1*], Atsushi Sugiyama[2,3,4], Yoshinobu Oshikiri[5], Ryoichi Morimoto[6], Iwao Mogi[7], Miki Miura[8], Satoshi Takagi[9], Jeonghun Kim[10], Yusuke Yamauchi[4,10**], Ryoichi Aogaki[4, 11***]

1 Hokkaido Polytechnic College, Otaru, Hokkaido 047-0292, Japan

2 Yoshino Denka Kogyo, Inc., Yoshikawa, Saitama 342-0008, Japan

3 Research Organization for Nano and Life Innovation, Waseda University, Shinjuku, Tokyo 162-0041, Japan

4 International Center for Materials Nanoarchitectonics (WPI-MANA), National Institute for Materials Science (NIMS), Tsukuba, Ibaraki 305-0044, Japan

5 Yamagata College of Industry and Technology, Matsuei, Yamagata 990-2473, Japan

6 Saitama Industrial Technology Center, Kawaguchi, Saitama 333-0844, Japan

7 Institute for Materials Research, Tohoku University, Aoba, Sendai 980-8577, Japan.

8 Polytechnic Center Kimitsu, Kimitsu, Chiba 299-1142, Japan

9 Graduate School of Symbiotic Systems Science and Technology, Fukushima University, Fukushima 960-1296, Japan

10 School of Chemical Engineering and Australian Institute for Bioengineering and Nanotechnology (AIBN), The University of Queensland, Brisbane, QLD 4072, Australia

11 Polytechnic University, Sumida, Tokyo 130-0026, Japan

E-mails:

* miura@hokkaido-pc.ac.jp

** YAMAUCHI.Yusuke@nims.go.jp

*** AOGAKI.Ryoichi@nims.go.jp

Supplement A. (References follow the manuscript)

Thermodynamic foundations. A system of electrolytic cell composed of MHDE and electrolytic solution is firstly introduced. As shown in Fig. A1, it is divided into two parts; one is a subsystem including electrode reactions (A), and the other is the environment (B). The system is replaced by an equivalent circuit (Fig. A2); the cell is connected in a series with an infinitely high resistance and a potentiostat, so that the cell voltage is kept in zero-current cell potential, i.e., electromotive force (*emf*), U, which has the following relationship with the Gibbs free energy of the cell reaction.

$$\Delta_R G_{CR} = -nFU \qquad \text{(A1)}$$

where n is the positive charge number transferring in the cell reaction, F is Fadaday constant (96500 C mol^{-1}), and $\Delta_R G_{CR}$ is the difference of the Gibbs free energy between the product and reactant of the cell reaction. The potentiostat can change the Gibbs free energy of the free electron participating the reaction, $\Delta_R G_e$, which is the free energy difference of electrons between the cathode and anode. In view of the negative charge of electron, we have

$$\Delta_R G_e = nFV \qquad \text{(A2)}$$

where V is the cell potential provided by the potentiostat.

Ionic vacancy is produced by the cell reaction. However, since there is no entropy production in the solvation of ionic vacancy [7], the Gibbs free energy for the vacancy solvation is equal to zero. At the same time, transferring electron supplies the Boltzmann energy and the polarization energy of the initial embryo vacancy core to ionic vacancy, losing the same amount of kinetic energy, so that the total difference of the Gibbs free energy in the circuit is expressed by $\Delta_R G_{CR}$ and $\Delta_R G_e$.

$$\Delta_R G_R = \Delta_R G_{CR} + \Delta_R G_e = -nF(U - V) \qquad \text{(A3)}$$

As a result, the total enthalpy difference in the cell reaction $\Delta_R H_R$ is written by the $\Delta_R G_R$ and the total entropy difference $\Delta_R S_R$ at a temperature T as follows,

$$\Delta_R H_R = \Delta_R G_R + T\Delta_R S_R \qquad \text{(A4)}$$

When the cell potential is equal to the *emf,* i.e., $V = U$, the difference of the Gibbs free energy becomes zero, $\Delta_R G_R = 0$, so that Eq. (A4) yields the reversible relationship.

$$\Delta_R H_R = T\Delta_R S_R (= Q) \qquad \text{(A5)}$$

Namely, the difference of the enthalpy is equal to the heat Q transferring between the subsystem and the environment.

The reversible heat generated at the electrode/electrolyte junction by electrochemical reaction arises from the change in partial molar entropy of the half-cell reaction plus terms which relate to the interaction between heat and mass transport in the electrode (i.e., the thermocouple or electronic Seebeck effect) and the electrolyte (i.e., the thermal diffusion or Soret effect) [25, 26]. However, in the present case, the electronic

transport-related terms are cancelled since the same electrode and metal lead are used in each half cell [27], and due to the isothermal condition maintained by MHD flow, the electrolytic transport-related terms are disregarded. Thus, the reversible heat for the whole cell reaction can be calculated simply from the entropy change of reaction, i.e., $T\Delta_R S_R$ [13]. Furthermore, the half cell reactions in Eqs. (1) and (2) nullify their reversible heats, so that $T\Delta_R S_R = 0$ is concluded. Namely, if neglecting vacancy annihilation, in the present system, heat generation except for Joule's heat would not be observed.

Equation of energy conservation. Then, the resistance R_∞ is removed, so that depending on the value of V (V), an electrolytic current I (A) flows in the subsystem, yielding the kinetic energy of the MHD flow, the dissipative heat by viscosity and the work by pressure. Here, due to mixing of the solution by the MHD flow, we can assume that the subsystem and the environment are kept isothermal, i.e., at the same temperature T.

According to the previous paper [22], as shown in Fig. A3, a two-dimensional MHD flow is divided into two parts, i.e., a main flow (Domain D_1) and two boundary layers (Domain D_2). The former occupies the central major part of the MHD flow, which behaves as an inviscid flow. The kinetic energy is generated by the work of Lorentz force, so that we can estimate it in the following,

$$\int_{D_1} \frac{1}{2}\rho \vec{u}^2 dv = \int_{D_1} \gamma B_z \langle i \rangle L dv \qquad \text{(A6)}$$

where ρ is the density of the solution, $\vec{u}$ is the velocity of the main flow, D_1 is the domain enclosing the main flow shown in Fig. A3, and dv is the volume element. γ is the cell constant, B_z is the applied magnetic flux density in the z-direction and $\langle i \rangle$ is the average current density and L is the electrode length. In view of two-dimensional flow, by taking a unit length in z-direction, the right hand side of Eq. (A6) is simply rewritten by

$$\int_{D_1} \gamma B_z \langle i \rangle L dv = \gamma B_z I L w \qquad \text{(A7)}$$

where I is the total current and w is the main flow width. Using the data, $\gamma \approx 1.0$, $B_z \approx 10$ T, $I \approx 10^{-1}$ A, $L \approx 10^{-2}$ m and $w \approx 10^{-3}$ m, we can estimate the kinetic energy production in the subsystem,

$$\int_{D_1} \frac{1}{2}\rho \vec{u}^2 dv \approx 10^{-5}\ \mathrm{J\ s^{-1}} \qquad \text{(A8)}$$

On the other hand, as will be discussed later, the irreversible heat production in the electrode reactions is estimated by the following Joule's heat production, which is calculated by $(V-U) \approx 10^{-1}$ V and $I \approx 10^{-1}$ A.

$$(V-U)I \approx 10^{-2}\ \mathrm{J\ s^{-1}} \qquad \text{(A9)}$$

Namely, in the category of thermodynamics, the kinetic energy production by the MHD flow can be disregarded.

On the contrary to the main flow, the boundary layer is a viscous flow, generating dissipative heat. Such dissipation represents the irreversible conversion of mechanical energy to thermal energy due to the action of fluid stress, which is calculated by dissipation function Φ [28]. Φ is the dissipation of energy per unit time per unit volume. In the boundary layers shown in Fig. A3, the x-component of the velocity u^* is dominant, and the variation in the y-direction is prevailing, so that Φ is estimated by

$$\Phi \approx \mu \left(\frac{\partial u^*}{\partial y} \right)^2 \tag{A10}$$

where μ is the viscosity. As a result, the dissipation from both boundary layers are

$$2\int_{D_2} \Phi dv \approx 2\mu \left(\frac{|\vec{u}|}{\delta} \right)^2 A\delta \tag{A11}$$

where each D2 is the domain enclosing a boundary layer, A is the surface area of the electrode, δ is the average boundary layer thickness and $\vec{u}$ is the velocity of the main flow. Using Eqs. (A6) and (A7), we have

$$|\vec{u}| = \sqrt{\frac{2\gamma B_z IL}{\rho A}} \tag{A12}$$

Substituting Eq. (A12) into Eq. (A11), we obtain

$$2\int_{D_2} \Phi dv \approx \frac{4\mu\gamma B_z IL}{\rho\delta} \tag{A13}$$

With the data ; $\mu \approx 10^{-3}$ Pa s (N m^{-2}s), $\gamma \approx 1.0$, $B_z \approx 10$ T, $I \approx 10^{-1}$A, $L \approx 10^{-2}$ m, $\rho \approx 10^3$ Kgm^{-3}, and $\delta \approx 10^{-3}$m, we can estimate Eq. (A13) as follows.

$$2\int_{D_2} \Phi dv \approx 10^{-5} \text{ J s}^{-1} \tag{A14}$$

Comparing the Joule heat production 10^{-2}J s^{-1} in Eq. (A9) with the heat dissipation 10^{-5}J s^{-1} in Eq. (A14), we can neglect the effect of the dissipation. Finally, since the pressure difference ΔP between the inlet and outlet of the MHD electrode with two open ends is equalized to zero [22], the remaining part of the stress work of the pressure in the boundary layers that goes into enthalpy is also disregarded.

- Fig. A3 -

As a result, the energy conservation in the subsystem is expressed by the reaction enthalpy H_R, as follows.

$$\frac{dH_R}{dt} = \Delta_R H_R \frac{I}{nF} \tag{A15}$$

In the environment, the ionic vacancies with opposite charges are mixed to collide with each other, emitting their reaction heat. In addition, considering that heat enters from or goes to the outside of the environment, as shown in Fig. A4, we obtain the energy conservation equation in the electrode system.

$$\frac{d}{dt}\left(H_{en}+H_R\right)=\gamma_{col}Q_{ann}\frac{|I|}{nF}+h_s\left(T_a-T\right) \qquad \text{(A16)}$$

where H_{en} and H_R are the enthalpies created in the environment and the subsystem, respectively. Q_{ann} is the molar excess heat (J mol^{-1}) arising from the pair annihilation by the collision of the ionic vacancies, and γ_{col} is the collision efficiency, i.e., $\gamma_{col}Q_{ann}$ is the observed molar excess heat. The total heat per unit time entering the system is expressed by the production of the integral heat-transfer coefficient h_s (JK^{-1} s^{-1}) and the temperature difference T_a-T, where T_a and T are the ambient temperature and the system temperature, respectively. Ionic vacancies are always created unrelatedly to the sign of electrolytic current, so that the absolute value of the electrolytic current $|I|$ is introduced.

- Fig. A4 –

Substituting Eq. (A4) into Eq. (A15), and inserting the resulting equation into Eq. (A16), we have

$$\frac{dH_{en}}{dt}=-\frac{1}{nF}\left(\Delta_R G_R+T\Delta_R S_R\right)I+\frac{1}{nF}\gamma_{col}Q_{ann}|I|+h_s\left(T_a-T\right) \qquad \text{(A17)}$$

As shown in Fig. A4, in the environment, there is no non-expansion work, i.e., no change in Gibbs free energy, which, as indicated in Eq. (A5), leads to the heat generation by the enthalpy H_{en}, i.e.,

$$\frac{dH_{en}}{dt}=C_{sys}\frac{dT}{dt} \qquad \text{(A18)}$$

where C_{sys} (J K^{-1}) implies the system heat capacity called calorimeter constant. Substituting Eqs. (A3) and (A18) into Eq. (A17), we finally obtain

$$C_{sys}\frac{dT}{dt}=\Delta VI-\frac{T\Delta_R S_R}{nF}I+\frac{\gamma_{col}Q_{ann}}{nF}|I|+h_s\left(T_a-T\right) \qquad \text{(A19)}$$

where ΔV is the overpotential applied to the cell (cell voltage), i.e.,

$$\Delta V\equiv U-V \qquad \text{(A20)}$$

ΔV can be regarded as the potential loss due to the overpotentials and ohmic potential drop, although they may result from complicated reaction-diffusion processes. The product ΔVI is the irreversible heat generation term, which is always positive ; ΔV changes sign whenever I changes sign.

$-T\Delta_R S_R I/nF$ in Eq. (A19) is the reversible heat-generation term and is directly related to the enthalpy change due to the electrochemical reaction. It changes sign when I changes sign.

The term $\gamma_{col}Q_{ann}|I|/nF$ is the heat generation from the vacancy collision, and is always positive because ionic vacancies are produced unrelatedly to the sign of *I*. Then, the term $h_s(T_a - T)$ is the heat entering to or leaving from the whole cell system per unit time, of which sign is dependent of the temperature difference, $T_a - T$.

In the case where cathode and anode are made of the same material, there is no specific direction of current, so that we can define the signs of *I* and ΔV positive, i.e., Eq. (A19) is a little simplified as

$$C_{sys}\frac{dT}{dt} = \Delta VI + \frac{1}{nF}\{-T\Delta_R S_R + \gamma_{col}Q_{ann}\}I + h_s(T_a - T) \qquad \text{(A21)}$$

Heat compensation for a quasi-adiabatic system. Since the cell system is non-adiabatic, during experiments, heat is always going into or leaving from it. For compensating the heat transfer, we must determine the transferring heat amount. At the end of experiment, after switching off the electrolytic current *I,* heat generation ceases, and the temperature of the heated system starts to decrease with time. Equation (A21) is thus reduced to

$$C_{sys}\frac{d\Delta T}{dt} = -h_s \Delta T \qquad \text{(A22)}$$

where ΔT is the temperature difference, defined by the solution temperature *T* and the ambient temperature T_a.

$$\Delta T \equiv T - T_a \qquad \text{(A23)}$$

Equation (A22) is integrated from *t* = 0 to *t* = *t*. so that we have

$$\Delta T = \Delta T_0 \exp(-\alpha t) \qquad \text{(A24)}$$

where ΔT_0 is the initial temperature difference, and α is the time constant (s^{-1}) of escaping heat, i.e.,

$$\alpha \equiv h_s / C_{sys} \qquad \text{(A25)}$$

It should be noted that Eq. (A24) is effective only when the electrode system is isothermal, defined by a single temperature *T*, which can be monitored by the thermo-sensors set in the system.

By compensating the escaping heat, we obtain the temperature difference in an adiabatic system. In Eq. (A21), the escaping heat term is transferred from the right hand side to the left hand side, and the heat production $C_{sys}\Delta T^*$ in the quasi-adiabatic system is defined by

$$C_{sys}\Delta T^* = C_{sys}\Delta T + h_s \int_0^t \Delta T dt \qquad \text{(A26)}$$

where ΔT^* is the temperature difference in the adiabatic system, calculated by

$$\Delta T^* = \Delta T + \alpha \int_0^t \Delta T dt \qquad \text{(A27)}$$

Namely, with the compensated ΔT^*, Eq. (A21) is converted to the following equation for an adiabatic system.

$$C_{sys}\frac{d\Delta T^*}{dt} = \Delta VI + \frac{1}{nF}\{-T\Delta_R S_R + \gamma_{col}Q_{ann}\}I \qquad \text{(A28)}$$

where ΔV and I are the the cell voltage (V) and the electrolytic current (A) of the MHDE, respectively, which are defined positive. n is the positive charge number tranferring in the cell reaction, F is Fadaday constant (96500 C mol^{-1}). $\Delta_R S_R$ is the molar entropy production (J K^{-1}mol^{-1}) by the cell reaction neglecting vacancy production.

Figures

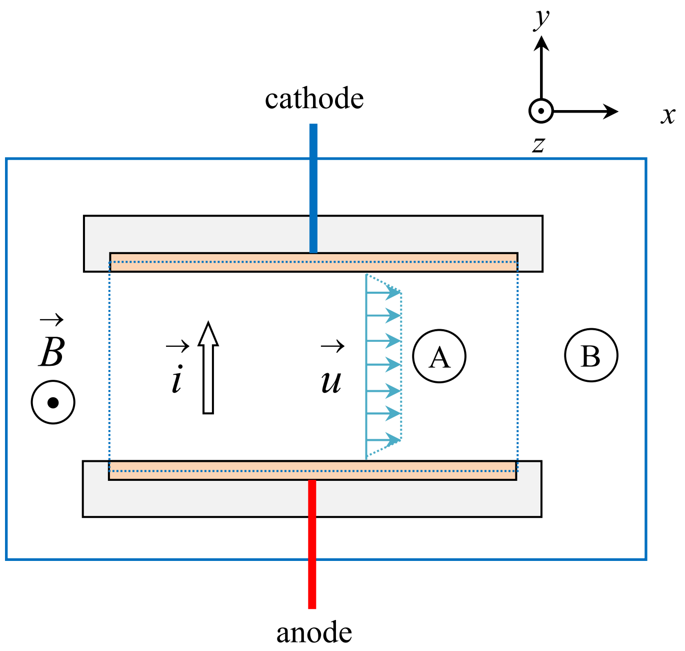

Figure A1 | MHD electrode system.

Ⓐ, Subsystem of electrode reaction; Ⓑ, Environment; $\vec{B}$, Magnetic flux density; $\vec{i}$, Electrolytic current density; $\vec{u}$, Fluid velocity.

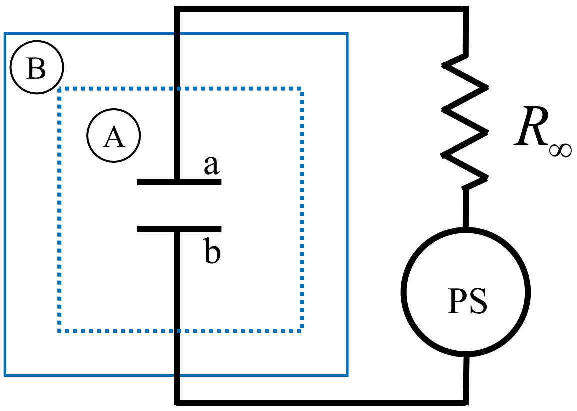

Figure A2 | Circuit diagram of the subsystem.
Ⓐ, Subsystem; Ⓑ, Environment ; a, Cathode ; b, Anode ; R_∞ , Infinitely high resistance; PS, Potentiostat.

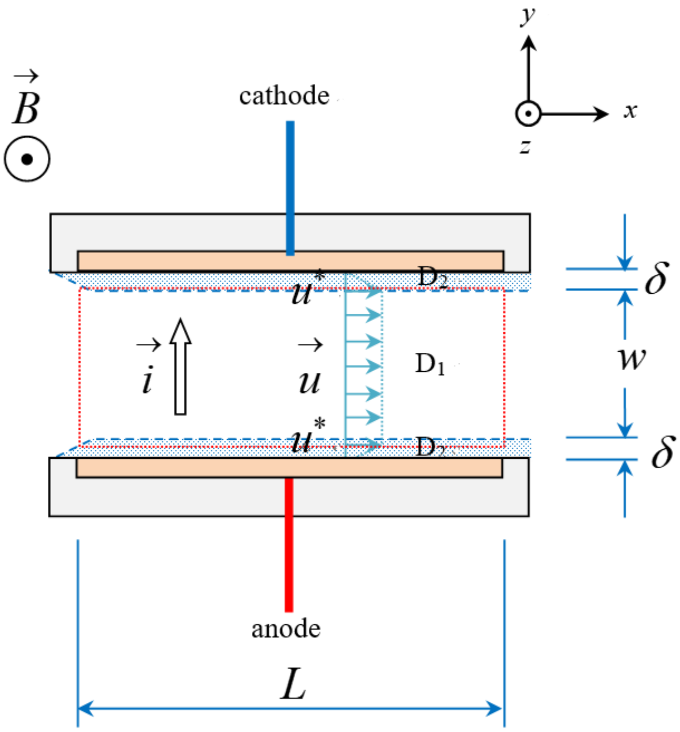

Figure A3 | Domain D_1 and domain D_2 defined by the mainflow and boundary layers.

D_1, Domain enclosing the main flow; D_2, Domain enclosing the boundary flow; L, Electrode length; w, Main flow width; δ, Boundary layer thickness; $\vec{B}$, Magnetic flux density; $\vec{i}$, Electrolytic current density; $\vec{u}$, Fluid velocity of the main flow; u^* , Fluid velocity of the boundary layer.

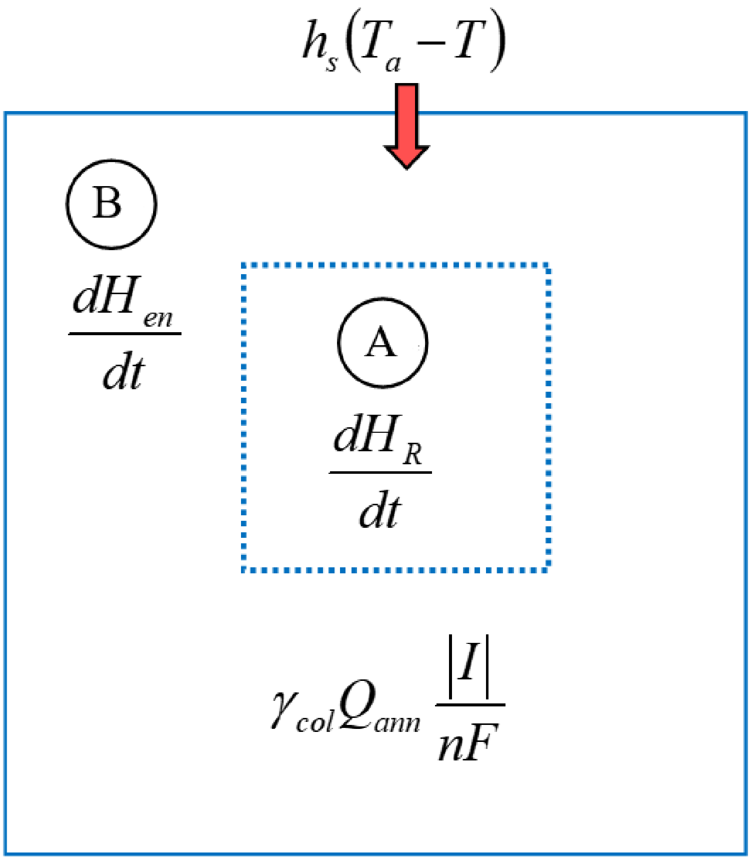

Figure A4 | Energy conservation of the whole system.

Ⓐ**,** Subsystem; Ⓑ, Environment; H_{en}, Enthalpy of the environment; H_R, Enthalpy of the subsystem; $\gamma_{col}Q_{ann}$, Observed molar excess heat of ionic vacancy; h_s, Integral heat-transfer coefficient ; T, System temperature; T_a, Ambient temperature.
